# Genome-Wide Gene Expression Analysis of *Bordetella pertussis* Isolates Associated with a Resurgence in Pertussis: Elucidation of Factors Involved in the Increased Fitness of Epidemic Strains

**DOI:** 10.1371/journal.pone.0066150

**Published:** 2013-06-11

**Authors:** Audrey J. King, Saskia van der Lee, Archena Mohangoo, Marjolein van Gent, Arno van der Ark, Bas van de Waterbeemd

**Affiliations:** 1 National Institute for Public Health and the Environment (RIVM), Laboratory for Infectious Diseases and Screening (LIS) Centre for Infectious Disease Control, Bilthoven, The Netherlands; 2 National Institute for Public Health and the Environment (RIVM), Department of Vaccinology, Bilthoven, The Netherlands; Universidad Nacional de La Plata., Argentina

## Abstract

*Bordetella pertussis (B. pertussis)* is the causative agent of whooping cough, which is a highly contagious disease in the human respiratory tract. Despite vaccination since the 1950s, pertussis remains the most prevalent vaccine-preventable disease in developed countries. A recent resurgence pertussis is associated with the expansion of *B. pertussis* strains with a novel allele for the pertussis toxin (ptx) promoter *ptxP3* in place of resident *ptxP1* strains. The recent expansion of *ptxP3* strains suggests that these strains carry mutations that have increased their fitness. Compared to the *ptxP1* strains, *ptxP3* strains produce more Ptx, which results in increased virulence and immune suppression. In this study, we investigated the contribution of gene expression changes of various genes on the increased fitness of the *ptxP3* strains. Using genome-wide gene expression profiling, we show that several virulence genes had higher expression levels in the *ptxP3* strains compared to the *ptxP1* strains. We provide the first evidence that wildtype *ptxP3* strains are better colonizers in an intranasal mouse infection model. This study shows that the *ptxP3* mutation and the genetic background of *ptxP3* strains affect fitness by contributing to the ability to colonize in a mouse infection model. These results show that the genetic background of *ptxP3* strains with a higher expression of virulence genes contribute to increased fitness.

## Introduction

*Bordetella pertussis* is a human-specific pathogen and the causative agent of whooping cough, or pertussis, which is an acute respiratory disease that is particularly severe in infants. Universal immunization programs have contributed to a significant reduction in the incidence of serious disease and mortality caused by *B. pertussis* [1], especially in infants and children. However, pertussis remains one of the leading causes of vaccine-preventable deaths worldwide despite extensive immunization [2], [3]. A resurgence in pertussis in recent years has been observed in highly immunized populations. Several causes for the re-emergence of pertussis have been suggested, including waning immunity and pathogen adaptation [2], [4] [5], [6]. Polymorphisms in *B. pertussis* surface proteins have been detected in several countries. Variations in the *B. pertussis* proteins pertussis toxin (Ptx) and pertactin (Prn) have been shown to affect vaccine efficacy in a mouse model [7] [8], [9] [9], [10]. Since the 1990s, strains with a novel allele for the Ptx promoter (*ptxP3*) associated with recent epidemics in the Netherlands have emerged in several European countries, replacing resident *ptxP1* strains [11], [12]. In the last 20 years, *ptxP1* and *ptxP3* have been predominating in the Dutch *B. pertussis* population. The recent expansion of *ptxP3* strains in the Netherlands and other countries in Europe, Asia and North and South America suggests that *ptxP3* strains carry mutations that have increased their fitness. Laboratory data has shown an increase in Ptx production in *ptxP3* strains, and epidemiological data has suggested that the *ptxP3* strains are more virulent [13] [14] than the *ptxP1* strains.

The expression of *B. pertussis* virulence factors is controlled by the two-component BvgAS sensory transduction system [1], [15], [16]. BvgAS controls the expression of a spectrum of phenotypic phases transitioning between a virulent (Bvg+) phase and a nonvirulent (Bvg-) phase, which is referred to as phenotypic modulation [17]–[20]. During the virulent Bvg+ phase, the BvgAS system controls the expression of over 100 virulence genes [21] by binding phosphorylated BvgA to the promoters of the virulence genes. Many of these genes are part of the core regulon defined by Cummings et al. [15]. The virulence core regulon consists of a set of 56 genes that are strongly upregulated in several different *B. pertussis* strains under virulent growth conditions. These genes include pertactin, pertussis toxin, filamentous hemagglutinin (FHA), fimbriae, adenlyate cyclase toxin, dermonecrotic toxin and the type III secretion system (TTSS) [15], [22]. Differences in Bvg-regulated gene expression between Bordetella species prompted the examination of differential regulation among isolates of the same species. Differential gene expression patterns within a species may reflect ongoing microevolution and could lead to better insight into the mechanisms of host adaptation. Diversification of gene expression profiles may also influence pathogenicity, which has been suggested in *Mycobacterium tuberculosis* [23].

A recent microarray-based analysis of the gene content of over 170 *B. pertussis* clinical strains isolated in different countries revealed genomic differences between the strains. Genes present in all of the isolates (core genes) are assumed to be phylogenetically conserved, while the genes that are variably present (variable genes) are proposed to be horizontally acquired or differentially lost within the species. The core and variable genome has been defined for *B. pertussis* based on microarray studies [15], [24], [25]. A strong correlation was found between the *ptxP* type and the gene content, suggesting that strains with different *ptxP* types form different lineages [24]. A similar analysis previously revealed that the *ptxP1* and *ptxP3* lineages are distinguished by a region of 18 genes present in *ptxP1* strains, but absent in all of the *ptxP3* strains that have been analyzed to date [26]. More recently, several sequencing and single nucleotide polymorphisms (SNP)-based studies have confirmed that *ptxP3* isolates are grouped together and form a separate branch [27], [28] [29]. To our knowledge, it is not known how the *B. pertussis ptxP3* strains differ in other key biological properties from the *ptxP1* strains, but it is suggested that the *ptxP3* strains are the fitter variants of *B. pertussis* [24], [27], [29].

While variation in genetic content is likely to be relevant in pathogenesis, differential gene expression may also be important. Gene expression profiling using DNA microarray technology provides a fingerprint of the full transcriptome, which allows a detailed comparison of strain-specific differences. In this study, we used genome-wide gene expression profiling, allelic exchange and a murine model of infection to gain insight into the factors involved in the global spread of the *ptxP3* strains.

## Results

### Growth and Strains

In a previous study, we analyzed the gene content of *B. pertussis* strains from different countries isolated between 1949 and 2008 using microarray-based CGH and found a strong correlation between the gene content and the *ptxP* allele [24]. In this study, we analyzed the genome-wide microarray-based gene expression of a subset of these strains to evaluate the differences in expression between the *B. pertussis* strains isolated in the Netherlands between 1949 and 2008 carrying a *ptxP1* (n = 9) or *ptxP3* allele (n = 5). Strains were grown under Bvg+ conditions in a chemically defined growth medium [30], [31] that provides highly reproducible gene expression and bacterial growth results [32]. Triplicate shake flask cultivations (independent cultures) were grown for each strain. The strains were monitored spectrophotometrically at an optical density of 590 nm (OD_590_) to confirm logarithmic growth (data not shown). Changes in growth phase-associated gene expression were previously studied using transcriptional profiling of vaccine strain 509 under similar conditions [32]. Based on these and other unpublished data (personal communication King *et al.*), the optimal harvest point for the RNA samples was estimated at OD = 0.4+/−0.05. This time point was chosen to minimize the specific effects of the growth phase.

### Transcriptomic Analysis of *B. pertussis* Strains

Microarray-based gene expression was measured using the previously described pan-*Bordetella* microarray based on the sequence of *B. pertussis* (Tohama I strain) and all of the extra genes found in *B. parapertussis* strain 12822 and *B. bronchiseptica* strain RB50 [24]. Genome-wide transcriptional profiles of the *B. pertussis* strains carrying the *ptxP1* allele (n = 9) and *ptxP3* allele (n = 5) were determined to identify differentially regulated genes. The transcript levels were assessed in all (at least) -three biological replicates of each strain (Table S1). Microarray data of the 3714 genes present in all of the strains analyzed in this study were used. Data analysis revealed significant differential gene expression in 975 genes between both groups of strains (*ptxP1* and *ptxP3*; false discovery rate (FDR) <0.05; corresponds to P<0.013117). The transcription of 952 genes was significantly upregulated in *ptxP3* strains, with 818 core genes. For 134 variable genes, expression was significantly higher in the *ptxP3* strains. The transcription of 23 genes was significantly downregulated in the *ptxP3* strains, with 19 core genes (Table 1). Four variable genes were downregulated in the *ptxP3* strains. A summary of all of the significantly regulated genes is provided in Table S2.

**Table 1:** Differentially expressed genes in *ptxP3* vs. *ptxP1 Bordetella pertussis* strains.

	*ptxP3* vs. *ptxP1*	Core genes	Variable genes
Total number of genes expressed that were significantly (FDR<0.05) different	975	837	138
Number of upregulated genes in P3 vs P1	952	818	134
Number of downregulated genes in P3 vs P1	23	19	4

### Functional Analysis

**Figure 1:**
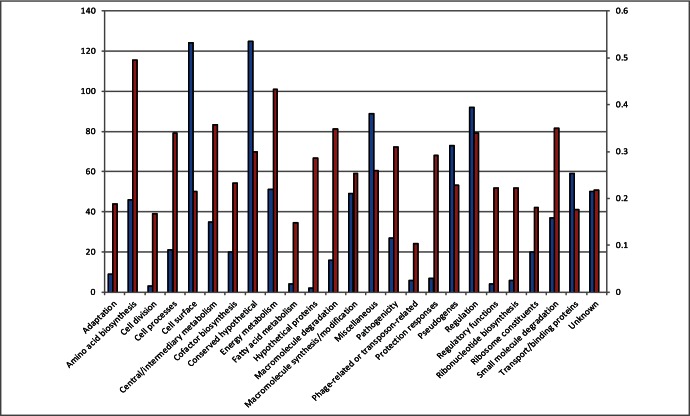
Functional categories of differentially expressed genes between the *ptxP1* and *ptxP3* strains. The gene count (absolute number of genes) of differentially expressed genes per category (blue). And the gene count of differentially expressed genes relative to the total number of genes (red) present in the genomes of the analyzed strains.

The proportion of various functional categories of the differentially expressed genes in the *ptxP3* and the *ptxP1* strains were determined. The functional categories classified by Parkhill et al. [33] were used with modification in genes involved in pathogenicity [34]. Differentially expressed genes were detected in several functional categories. There were 125 conserved hypothetical genes differentially expressed in *ptxP3* compared to *ptxP1*, followed by cell surface genes, regulation genes, miscellaneous genes and pseudogenes (Figure 1). Differences in expression relative to the total number of genes present in the strains in the amino acid biosynthesis, energy metabolism, regulation and central/intermediary metabolism categories were significantly increased (P<0.05) in all of the genes differentially expressed between *ptxP3* and *ptxP1* (Figure 1).

Several virulence-associated genes were differentially expressed in the *ptxP3* strains. Of the 57 genes belonging to the virulence core regulon, which is described as the genes expressed in all *B. pertussis* strains when grown under conditions that promote virulence (genes implicated in *B. pertussis* virulence) [15], [22], [35], about 21 (37%) (Table 2) had a significantly higher abundance of transcripts in the *B. pertussis* strains carrying the *ptxP3* allele, suggesting that the *ptxP3* strains may be more virulent. The differences in expression between the *ptxP1* and *ptxP3* strains were significant even though they generally did not differ by more than a factor of 1.5-fold, with the exception of the, BP0500, BP1568, BP2252, BP2254 and BP2315 (Table 2). Interestingly, the virulence sensor protein (BvgS) that forms the BvgAS two component sensory regulatory system important in the regulation of Bordetella virulence was slightly, but significantly, upregulated in the *ptxP3* strains. The virulence factor transcriptional regulator (BvgA) also showed a slightly higher expression (P = 0.014172) in the *ptxP3* strains, but the difference was slightly above the FDR <0.05 cutoff. The microarray data showed a slightly higher (factor of 1.14–1.25-fold) expression of the genes in the ptx-operon. BP3783 (ptxA), BP3784 (ptxB), BP3785 (ptxD), BP3786 (ptxE) and BP3787 (ptxC), the difference in expression of ptxB in *ptxP3* and *ptxP1* strains with a 1.14 fold change P = 0.022 FDR = 0.07, slightly above the FDR cutoff of 0.05 and is there was not shown in Table 2. BP1119, the serotype 2 precursor was expressed significantly lower in *ptxP3 strains* (not shown).

**Table 2:** Genes from the vir-core regulon were significantly upregulated in the *ptxP3* strains compared to the *ptxP1* strains.

GeneID	annotation	symbol	Fold change in ptxP3 vs ptxP1	P-value	FDR
BP0216	autotransporter subtilisin-like protease	sphB1	1,120588003	0,010467976	0,0429118
BP0500	hypothetical protein	bteA, bopC	1,578536152	0,007383703	0,0338975
BP1119	serotype 2 fimbrial subunit precursor	fim2	−9,718882751	3,37339E−06	0,0002724
BP1201	tracheal colonization factor precursor	tcfA	1,379831085	6,31196E−05	0,0017365
BP1568	serotype 3 fimbrial subunit precursor	fim3	7,01726444	9,20698E−06	0,0004956
BP1877	virulence sensor protein	bvgS	1,186268148	0,002475936	0,0173685
BP1883	fimbrial adhesin	fimD, fhaE	1,244730773	5,81677E−05	0,0016601
BP2227	putative anti-sigma factor		1,269860344	0,005779169	0,0293623
BP2234	putative RNA polymerase sigma factor	brpL	1,266336408	0,002932043	0,0190378
BP2235	putative type III secretion protein	bscC	1,3146256	0,007375814	0,0338975
BP2252	putative outer protein B	bopB	1,58564765	0,009314957	0,0394478
BP2254	putative regulatory protein	bcrH1	1,553109473	0,01021466	0,0421139
BP2315	autotransporter	vag8	1,701057776	0,000587588	0,0066942
BP2924	putative exported protein		1,23341571	0,001204634	0,0108068
BP2925	conserved hypothetical protein		1,26736157	1,52744E−05	0,0006696
BP2927	putative integral membrane protein		1,393854682	5,92749E−07	8,467E−05
BP3783	pertussis toxin subunit 1 precursor	ptxA	1,173210514	0,008153995	0,0362248
BP3785	pertussis toxin subunit 4 precursor	ptxD	1,246241214	3,88345E−05	0,0012223
BP3786	pertussis toxin subunit 5 precursor	ptxE	1,203998944	0,000547153	0,0064512
BP3787	pertussis toxin subunit 3 precursor	ptxC	1,251857404	6,94186E−05	0,0018819
BP3795	putative bacterial secretion system protein		1,160210441	0,00751076	0,0341432
BP3796	putative bacterial secretion system protein		1,156985062	0,006232546	0,0308084

**Figure 2:**
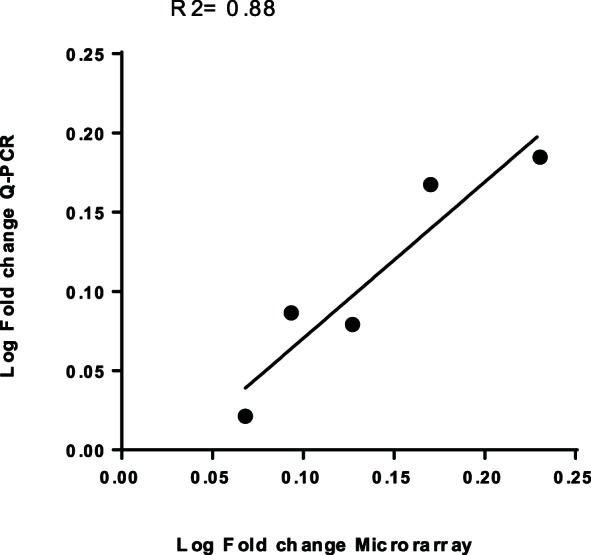
Comparison of gene expression measurements by microarray hybridization and quantitative real-time PCR. Log-transformed (in base 10) fold change values of the Q-PCR data (y-axis) were plotted against the log-transformed fold change values of microarray data (x-axis). The coefficient of determination (R 2) is given.

Microarray data were validated by real time quantitative PCR assays on a small selection, 5 (BVG controlled) genes (Table 3). Real time quantitative PCR analysis of this set was highly concordant with the microarray gene expression data r2 = 0.88 Figure 2.

**Table 3:** Relative differences in gene expression between *ptxP3* and *ptxP1* strains, as detected by microarrays and Q-PCR.

		MICROARRAY		Q-PCR	
GeneID	Protein Product	Fold Change P3/P1	P value P3 vs P1	Ratio P3/P1	P value P3 vs P1
BP2315	autotransporter	1,7	0,001	1,53	0,003
BP3405	outer membrane porin protein OmpQ	1,34	0,000	1,20	0,053
BP3494	serum resistance protein	1,48	0,000	1,47	0,022
BP3783	pertussis toxin subunit 1 precursor	1,17	0,008	1,05	0,500
BP3785	pertussis toxin subunit 4 precursor	1,24	0,000	1,22	0,263

### Colonization of *ptxP1* and *ptxP3 B. Pertussis* Strains in a Mouse Model

**Figure 3:**
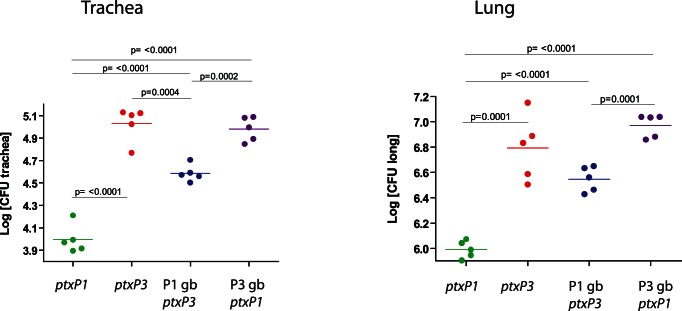
Role of the *ptxP3* and *ptxP1* mutations and the genetic background of *ptxP1* and *ptxP3* strains in colonizing the trachea (A) and lungs (B) in mice. Mice were intranasally infected with the wildtype *ptxP1* strain, the *ptxP3* strain, isogenic strains carrying the *ptxP3* allele in the *ptxP1* genetic background (P1 gb:*ptxP3*) or the *ptxP1* allele in the *ptxP3* genetic background (P3 gb:*ptxP1*). CFUs were determined in the trachea and lungs four days post-infection. The mean is indicated by a thin line. P-values (uncorrected for multiple tests) were shown if greater than 0.05. The experiment was performed two times representative result is shown.

To discriminate between the role of individual *ptxP3* and *ptxP1* mutations and the genetic background (gb) of the strains, we constructed two different isogenic strains that carry the *ptxP3* allele in the *ptxP1* genetic background (P1 gb:*ptxP3*) or the *ptxP1* allele in the *ptxP3* genetic background (P3 gb:*ptxP1*). The colonization of isogenic and wild type strains was tested in an intranasal mouse model that was previously described [16] [7]. Colonization was assessed four days after infection in the lungs and trachea of mice (Fig. 2). We demonstrate for the first time that wild type *ptxP3* strains colonize more efficiently than the wild type *ptxP1* strains in the lungs and trachea of mice (P<0.0001). The P3 gb:*ptxP1* strains and wild type *ptxP3* strains showed similar colonization levels in the lungs and trachea, suggesting that the genetic background of the strain significantly contributes to the observed differences. The P1 gb:*ptxP3* strains showed increased colonization in the lungs and trachea than the wild type *ptxP1* strains (P<0.0001), which suggests that the *ptxP3* allele contributes to the increased colonization. Colonization was decreased in the P1 *gb:ptxP3* strains compared to the wild type *ptxP3* strains, but the differences were not significantly different in lungs and trachea (Fig. 3). In conclusion, these results suggest that the *ptxP3* allele and the genetic background in which this allele is embedded enhance colonization in a mouse model.

## Discussion

Microarray analysis was used to evaluate differences in gene expression on a genome-wide scale between two different *B. pertussis* lineages. A previous study demonstrated that *B. pertussis* strains carrying the *ptxP3* allele differ from strains with the *ptxP1* allele in the *ptxP* promoter sequence and gene content based on microarray-based CGH analyses [24]. *PtxP3* strains have been associated with pertussis epidemics in several countries worldwide [22] [36], [37], and evidence has suggested that the *ptxP3* strains are more virulent [13]. Mooi *et al.* [38] showed that *ptxP3* strains produce a higher level of pertussis toxin compared to strains with the *ptxP1* allele, which may influence the fitness of these strains. In this study, we investigated differences between the *ptxP1* and *ptxP3* strains that could explain the success of the *ptxP3* strains. We compared the transcriptional profiles of *B. pertussis* strains with the *ptxP1* and *ptxP3* alleles on a genome-wide scale. Gene expression analysis revealed that many genes, including genes that are known to be involved in virulence (most notably the BvgS gene), were more highly expressed in the *ptxP3* strains. Previous studies have suggested that strains with different *ptxP* types form different lineages [24] [27]–[29]. This study provides the first evidence that a wildtype *B. pertussis* strain with the *ptxP3* allele is a better colonizer in the intranasal mouse infection model than a *B. pertussis* strain with a *ptxP*1 allele (Fig. 2). By using allelic exchange and subsequent analysis in the intranasal mouse infection model, we showed that not only the *ptxP3* allele is responsible for improved colonization and that the genetic background of *B. pertussis* strains also contributes to the success of the strain (Fig. 2).

Transcriptomic analysis and analysis of isogenic strains in a mouse model were used to gain insight into the biological properties of *B. pertussis* strains with different *ptxP* alleles, particularly *ptxP1* and *ptxP3*. Using transcriptomics, we attempted to identify new polymorphic loci important for adaptation and virulence. Because *ptxP3* strains are more successful [22] [36], [37] and possibly more virulent [13], we hypothesized that loci enhancing the virulence are more highly expressed in these strains. In a previous study by Mooi *et al.* [38], several arguments were raised that underlined the role of the *ptxP3* mutation in the success of these strains. We investigated whether various genes expressed at higher levels in *ptxP3* strains may contribute to the increased fitness of the *ptxP3* strains. The whole genome-level gene expression profiles of *B. pertussis* strains with either the *ptxP1* or *ptxP3* alleles revealed loci in the *B. pertussis* population that are differentially expressed between the strains with *ptxP1* and *ptxP3* alleles. The transcriptional profiles demonstrate that many genes (n = 952) are upregulated (FDR<0.05 and P<0.013117) in the *ptxP3* strains. Q-PCR data for a small selection of genes confirmed the higher expression in *ptxP3* strains compared to *ptxP1* strains, although in two genes the higher expression in p*txP3* strains by Q-PCR was not significantly different. The correlation between results by Q-PCR and hybridization with the microarray were shown to be very good for the 5 genes selected. Differentially expressed genes involved in amino acid biosynthesis (P = 0.00005), energy metabolism (P = 0.00049), and regulation (P = 0.00786) between the *ptxP1* and *ptxP3* strains were significantly overrepresented. Cummings *et al*. [15] described a set of genes that form the virulence core regulon. The genes in this regulon encode factors that are required for efficient infection and transmission [15]. Transcriptomic analyses of the *ptxP1* and *ptxP3* strains showed a 37% increase in expression of the virulence-associated genes that form the core regulon in the *ptxP3* strains, which indicates that these strains may be more virulent. Of the 21 genes with increased expression in the *ptxP3* strains, BP0500 (BteA alias bopC), BP1568 (serotype 3 precursor), BP2252, (bop B), BP2254 (bcrH 1) and BP2315 (vag 8) had expression levels that were more than 1.5-fold higher in the *ptxP3* strains. The virulence sensor protein (BvgS) was also slightly, but significantly, upregulated in the *ptxP3* strains. Han *et al.* [39] recently showed increased expression of BteA, which is thought to play a pivotal role in T3SS-mediated cell death [40]–[42] in non-vaccine type strains. BteA is known to be regulated by the BvgAS system. Our analysis showed increased expression of BteA in the *B. pertussis* strains carrying the *ptxP3* allele. Significantly increased expression of the *ptxP* operon was detected by microarray based gene expression even though the difference was less than 1.5-fold (Table 2). Our Q-PCR data showed slightly higher expression of two subunits of Ptx however in contrary to the microarray data this difference was not significant (Table 3). Previously Mooi et al. [38] had shown by using an ELISA technique that PtxS1 was expressed significantly higher in *ptxP3* strains compared to *ptxP1* strains. Ptx has been suggested to increase the severity of *B. pertussis* infections because the related *B. parapertussis*, which does not express Ptx, causes a less severe infection [43]. In summary, transcriptomic analyses revealed increased expression of several virulence-associated genes. The increased expression of these genes is likely to influence the virulence potential of the *ptxP3* strains. The expression of BP1119, the serotype 2 precursor is significantly lower in *ptxP3* strains.

The biological properties of wild type *ptxP1* and *ptxP3* strains were evaluated *in vivo* using an intranasal mouse infection model to determine the colonization ability of both strains in the trachea and lungs of mice (Fig. 2). We demonstrate for the first time significantly higher colonization of the wildtype *ptxP3* strain (P<0.0001) in the trachea and lungs of mice compared to the wild type *ptxP1* strain. Multivariate analyses performed by van Gent *et al.* previously showed that *ptxP* contributed to differences in colonization [44]. Because *ptxP1* and *ptxP3* strains form separate lineages as shown by differences in genetic content and DNA sequencing and SNP studies [26] [27], [28] [29], the contribution of genetic background was investigated in this study. To discriminate between the role of *ptxP3* and *ptxP1* mutations individually and the genetic background (gb) of the strains, two different isogenic strains were constructed carrying the *ptxP3* allele in the *ptxP1* genetic background (P1 gb:*ptxP3*) or the *ptxP1* allele in the *ptxP3* genetic background (P3 gb:*ptxP1*). In the mouse model, the strain with the *ptxP3* genetic background, but not the *ptxP3* allele itself (P3 gb: *ptxP1*), had a similar colonization ability as the wildtype *ptxP3* strain. A significantly higher colonizing ability compared to wildtype *ptxP1* strains was observed, suggesting that the genetic background of the *ptxP3* strains plays a role in increasing the ability to colonize. Since the colonization ability was similar to the wildtype *ptxP3* strain this result may also be interpreted in that the *ptxP3* allele itself does not contribute that much to the higher colonizing ablility.In contrast, colonization with the strain with the *ptxP3* allele in the genetic background of a *ptxP1* strain (P1 gb: *ptxP3*) showed significantly increased colonization compared to the wildtype *ptxP1* strain, which indicates that the *ptxP3* allele itself also contributes to increased colonization in the mouse model. To our knowledge, no data on the effects of the genetic background of *ptxP3* strains on colonizing ability have been previously published.

The higher colonization of *ptxP3* strains in the lungs and trachea of mice is in agreement with epidemiological data. Mooi et al. [38] suggested that expansion of the *ptxP3* strains in many countries worldwide indicates that *ptxP3* increases the fitness of the strain or is linked to other genetic loci that do. These researchers suggest that the *ptxP3* mutation confers increased fitness. A study in Sweden also suggested that *B. pertussis* strains differ in virulence because patients infected with strains of the PFGE profile BPSR11 were hospitalized for longer periods of time [14]. The results of the present study support earlier indications of increased fitness in *ptxP3* strains. We demonstrate that the *ptxP3* allele and the genetic background of the strains contribute to increased fitness.

## Materials and Methods

### Bacterial Strains and Seedlot Preparation

The *Bordetella pertussis* strains used in this study are listed in Table 4. To improve the reproducibility of growth and gene expression, frozen working seedlots were prepared in a chemically defined medium with a standardized protocol. The starting materials were vials containing 1 ml Verwey medium (RIVM, Bilthoven, Netherlands; chemically undefined) with 20% glycerol and varying OD_590_ stored at −80°C. One vial of each strain was used to inoculate a primary 500 ml shake flask containing 200 ml THIJS medium [30], [31] consisting of basic medium and a supplement (1% v/v) that was added to the basic medium shortly before inoculation. Shake flasks were incubated at 35°C on an orbital shaker at 200 RPM. When an OD_590_ of 1.0±0.2 was reached, a secondary shake flask was inoculated with 10 ml of the culture at OD_590_ = 1.0. For shake flasks at other optical densities, the volumes were adjusted to ensure that an equal amount of cells were used to inoculate each secondary shake flask. Secondary shake flasks were incubated at 35°C and 200 RPM until an OD of 1.00±0.05 was reached. The cultures were mixed with glycerol (17% v/v), divided into 10 ml working seedlots and stored at −140°C.

**Table 4:** Characteristics of *ptxP1* and *ptxP3* strains used in this study.

KEY	SPECIES	Country of isolation	Year of isolation	PtxP	Fim3	Prn	PtxA	Fim2	MLST (PtxP-Fim3-Prn1)	serotype	Gene content type	Sequence Type	Strain used for Expression- (E) or Animal studies (A)
B0558	Bordetella pertussis	Netherlands	1949	1	1	1	2	1	111	3	21	27	E
B0602	Bordetella pertussis	Netherlands	1995	1	1	1	1	nd	111	3	1	nd	E and A
B0689	Bordetella pertussis	Netherlands	1982	1	1	1	1	nd	111	2,3	4	nd	E
B0777	Bordetella pertussis	Netherlands	1996	1	1	2	1	1	112	3	3	nd	E
B1213	Bordetella pertussis	Netherlands	1967	1	1	1	1	1	111	3	1	6	E
B1834	Bordetella pertussis	Netherlands	1999	1	1	2	1	1	112	3	12	33	E
B1878	Bordetella pertussis	Netherlands	2000	1	1	2	1	1	112	2	3	7	E
B3234	Bordetella pertussis	Netherlands	2008	1	1	2	1	1	112	2,3	1	7	E
B3265	Bordetella pertussis	Netherlands	2008	1	1	2	nd	1	112	2	3	nd	E
B0607	Bordetella pertussis	Netherlands	1995	3	1	2	1	1	312	3	2	nd	E
B1917	Bordetella pertussis	Netherlands	2000	3	2	2	1	1	322	3	2	13	E and A
B2973	Bordetella pertussis	Netherlands	1988	3	1	2	1	1	312	3	6	16	E
B3104	Bordetella pertussis	Netherlands	2007	3	1	2	1	1	312	3	39	11	E
B3183	Bordetella pertussis	Netherlands	2008	3	1	2	1	1	312	3	2	11	E
nd	not determined												

### Bacterial Cultivation

For each strain, a preculture was inoculated with 10 ml of the seedlot. Precultures were grown in 500 ml shake flasks containing 200 ml THIJS medium at 35°C on an orbital shaker at 200 RPM. When the preculture reached OD_590_ = 1.00±0.05, 3 secondary shake flasks were started for each strain. The triplicate cultivations were inoculated with 10 ml preculture and grown as described. The initial density was OD_590_ = 0.050±0.05 for all of the secondary cultivations. Separate samples were collected from each secondary shake flask for RNA isolation and consecutive microarray analysis when the OD_590_ was 0.4±0.05.

### RNA Isolation, Preparation of Labeled cDNA and Microarray Analysis

For fixation of the RNA expression profile, 1 volume of bacterial culture was mixed with 2 volumes of a *RNase* retarding solution [45]–[47]. For each microarray sample, 2.5 ml of the culture at OD_590_ = 1.0 was used. For samples at other optical densities, the volumes were adjusted such that an equal amount of cells was used for each sample. The samples were concentrated by centrifugation and treated with Tris-EDTA buffer containing 0.5 mg/ml lysozymes (Sigma-Aldrich, Zwijndrecht, The Netherlands) for 3 minutes. Total RNA was extracted with the SV Total RNA Isolation System (Promega Benelux, Leiden, The Netherlands) according to the manufacturer’s protocol. The nucleic acid concentration was adjusted by precipitation, and spectral analysis was used to determine the final nucleic acid concentration and purity. RNA integrity was confirmed with the Bioanalyzer RNA6000 Nano assay (Agilent Technologies, Amstelveen, The Netherlands) according to the manufacturer's protocol.

Custom pan-*Bordetella* microarrays were constructed using the 8 x 15K format developed by Agilent Technologies (Wilmington, Delaware, USA). The set of 5,910 60-mer oligonucleotides (60-mer) in which one oligonucleotide corresponds to one gene covered 94% of the genes in the three sequenced *Bordetella* strains, including *B. pertussis* Tohama I, *B. parapertussis* 12822 and *B. bronchiseptica* RB50. In addition, 98 control probes were included in the microarray, and all of the spots were printed in duplicate (non adjacent). User-defined probes were uploaded through the Agilent eArray Web portal (http://earray.chem.agilent.com/earray/). Additional details on microarray production are available through the ArrayExpress microarray data repository (accession number A-MEXP-1697).

A two-color hybridization format was used for microarray analysis. For each biological replicate, RNA extracted from each test strain was used to create Cy5-labeled cDNA, and the (common) reference sample containing equal amounts of RNA from all experimental samples was used to create Cy3-labeled cDNA. The use of a common reference across different cDNA microarray experiments improves the reproducibility of the hybridization signals and allows the gene expression levels from separate experiments to be compared. Total RNA samples were reverse transcribed to cDNA and labeled with Cy3/Cy5 dyes using the Chipshot Indirect Labeling kit (Promega Benelux) according to the manufacturer’s protocol with one modification. A total of 2 µl of random nonamer primer without oligo-dT primers was used per reaction to reverse transcribe the total RNA. For each hybridization, 300 ng Cy3-labeled cDNA and 300 ng Cy5-labeled cDNA were combined with 5 µl 10× blocking agent and 1 µl 25× fragmentation buffer in a total volume of 25 µl according to the manufacturer’s protocol (Agilent). Prior to loading on the microarray, the hybridization solution was heated for 3 minutes at 60°C. Microarray slides were hybridized and treated as described in the Agilent protocols for two-color microarray-based gene expression analysis. Microarray experiment details are also deposited at array express under accession number E-MTAB-1594.

For quantitative PCR the total RNA was treated with DNase I to remove contaminating DNA. The quality of the RNA was evaluated by Agilent Bioanalyzer and Naonodrop spectrophotometry. The RNA was reverse transcribed using random hexamers and oligo d(T). Samples were tested in 384 well format in duplicate with a no-RT control for each sample. Universal Human Reference RNA and the control sample were also tested in duplicate with a no-RT control. The samples were analyzed with 6 assays: 5 target assays and 1 endogenous control, as detailed below. All samples were amplified using the Applied Biosystems Prism® 7900 Sequence Detection System with standard cycling conditions. Primer sequences for the examined 6 genes are in Table S3. Samples were tested in duplicate. BP0015 was used as endogenous control. All relative quantification data were calculated using the delta delta CT method. The gene expression for *ptxP1* strains was calculated as mean +/− SD for 8 strains and the gene expression for *ptxP3* strains was calculated as mean +/− SD for 5 strains. The relative difference in expression were presented as the ratio of *ptxP3* strains to *ptxP1* strains.

### Microarray Data Mining

The hybridized slides were scanned at a 5 µm resolution using a ScanArray Gx plus microarray scanner (Perkin Elmer) equipped with ScanArrray express software. The images from Agilent pan-*Bordetella* microarrays were analyzed using ImaGene software (Biodiscovery, El Segundo, CA, USA). Individual arrays were internally normalized between the Cy3 and Cy5 channels by LOWESS normalization. A hybridization ratio logarithm [log2(Cy5/Cy3)] was calculated and the gene expression data were normalized to the expression of a BP0015 DNA-directed RNA polymerase beta chain in each microarray slide to compensate for variations between the slides. The normalized data were further processed using Microsoft Excel and TMEV software from the TM4 suite (TIGR). Biological replicates were analyzed separately for all of the strains. The average expression of each individual gene was calculated for all of the strains carrying the *ptxP1* and *ptxP3* alleles. P-values were calculated with one–way ANOVA statistical analysis (t-test). The fold change in the *ptxP3* and *ptxP1* strains was determined as follows: 2 ^∧^ (average of expression in *ptxP3* strains – average of expression in *ptxP1* strains), if this number, between the brackets, is greater then 0 if not then 1/2 ^∧^ (average of expression in *ptxP3* strains – average of expression in *ptxP1* strains. All microarray data have been deposited in Array express under accession number E-TAB-1594.

### Construction of Recombinant *B. Pertussis* Strains

To generate a *B. pertussis* strain with the *ptxP1* allele in the genetic background of the *ptxP3* strain (P3gb, *ptxP1*), we constructed a suicide vector pSS1129– *ptxP1* by PCR amplification of an internal fragment of the ptxP promoter from genomic strain B0213 (Tohama I) using the Xba1F (GCT CTA GAC GCT GCA GTC CAA GGC GGT CGT C) and EcoR1R (GGA ATT CAT CCC GTC TTC CCC TCT GCG TTT TGA TG) primers. The PCR product was cloned into pSS1129 using the Xba1 and EcoR1 restriction sites. Suicide vectors were introduced into *B. pertussis* (B1917, *ptxP3*) via conjugation using *E. coli* SM10 as a donor strain. *B. pertussis* mutants were selected based on growth on BG-agar containing appropriate antibiotics. Removal of the vector sequences was forced by growing the conjugates on BG agar plates with 300 µg/ml streptomycin. A *B. pertussis* strain with a *ptxP3* allele was constructed in the genetic background of a *ptxP1* strain (P1gb, *ptxP3*) in a similar manner. The suicide vector pSS1129-*ptxP3* was constructed and introduced in *B. pertussis* (B0602, *ptxP1*). Proper insertion was confirmed by sequencing. The gene content of all of the mutant *B. pertussis* strains was analyzed using microarray-based CGH analysis. The strains B1917, *ptxP3 and* B0602, *ptxP1* have the most common gene content type for *ptxP3* and *ptxP1* strain respectively and were therefore good respresentatives for these two groups.

### Mouse Infection Model

All animal experiments were conducted according to relevant national and international guidelines. Strains were grown on Bordet-Gengou plates and animal experiments were performed as previously described [44]. This study was agreed upon by the Committee on animal Experimentation of the Netherlands Vaccine Institute (DEC-NVI, Bilthoven, the Netherlands) under permit numbers 201000079 and 201000257. Animal handling in this study was carried out in accordance with relevant Dutch national legislation, including the 1997 Dutch Act on Animal Experimentation.

## Supporting Information

Table S1 **Microarray gene expression data for all strains analyzed in this study.** DNA microarray analysis was used to measure the mRNA levels in *ptxP3* strains compared to mRNA levels in *ptxP1* strains. Gene expression data were shown for all replicates for each individual strain (*ptxP1* strains with a green column head and *ptxP3* strains with red column head). The gene expression data were normalized to the expression of a BP0015 DNA-directed RNA polymerase beta chain in each microarray slide to compensate for variations between the slides. The average expression of each individual gene was calculated for all of the strains carrying the *ptxP1* and *ptxP3* alleles. P-values were calculated with one–way ANOVA statistical analysis (t-test), P-values lower than 0.05 were highlighted in green. The fold change in the *ptxP3* and *ptxP1* strains were calculated as described in materials and methods section. The false discovery rate (FDR) is shown.
(XLSX)

Table S2 **Summary of all genes significantly up or down regulated in strains carrying the** ***ptxP3*** **allele compared to strains with the** ***ptxP1*** **allele.** The gene ID, gene name and gene category are shown.
(XLS)

Table S3 **Quantitative PCR Primer sequences for genes by Q-PCR in this study**. Primer sequences for 6 assays: 5 target assays and 1 endogenous control are shown. The gene IDs for targeted genes are given in the first column.
(XLSX)
